# Antibacterial and Antihemolytic Activity of New Biomaterial Based on Glycyrrhizic Acid and Quercetin (GAQ) against *Staphylococcus aureus*

**DOI:** 10.3390/jfb14070368

**Published:** 2023-07-13

**Authors:** Ewa Olchowik-Grabarek, Krzysztof Czerkas, Alimjon Davletboevich Matchanov, Rahmat Sulton Esanov, Umarbek Davlatboevich Matchanov, Maria Zamaraeva, Szymon Sekowski

**Affiliations:** 1 Laboratory of Molecular Biophysics, Department of Microbiology and Biotechnology, Faculty of Biology, University of Bialystok, 15-254 Bialystok, Poland; kc1802@wp.pl (K.C.); m.zamaraeva@uwb.edu.pl (M.Z.); 2 Institute of Bioorganic Chemistry, Academy of Sciences of the Republic of Uzbekistan, Tashkent 100143, Uzbekistan; olim_0172@mail.ru (A.D.M.); esanovrahmat2836@gmail.com (R.S.E.); 3 National University of Uzbekistan, Tashkent 700174, Uzbekistan; 4 Institute of Chemistry of Plant Substances, Academy of Sciences of the Republic of Uzbekistan, Tashkent 100170, Uzbekistan; umarbek.2017.matchanov@gmail.com

**Keywords:** quercetin, glycyrrhizic acid, α-hemolysin, *Staphylococcus aureus*, erythrocytes, membrane fluidity

## Abstract

The goal of this study is to obtain and characterize the complex of quercetin with glycyrrhizic acid, which is known to serve as a drug delivery system. Quercetin is a flavonoid with a wide range of biological activities, including an antimicrobial effect. However, quercetin instability and low bioavailability that limits its use in medical practice makes it necessary to look for new nanoformulations of it. The formation of the GAQ complex (2:1) was confirmed by using UV and FT-IR spectroscopies. It was found that the GAQ exhibited antimicrobial and antihemolytical activities against *S. aureus* bacteria and its main virulent factor—α-hemolysin. The IC_50_ value for the antihemolytical effect of GAQ was 1.923 ± 0.255 µg/mL. Using a fluorescence method, we also showed that the GAQ bound tightly to the toxin that appears to underlie its antihemolytic activity. In addition, another mechanism of the antihemolytic activity of the GAQ against α-hemolysin was shown, namely, its ability to increase the rigidity of the outer layer of the erythrocyte membrane and thus inhibit the incorporation of α-hemolysin into the target cells, increasing their resistance to the toxin. Both of these effects of GAQ were observed at concentrations below the MIC value for *S. aureus* growth, indicating the potential of the complex as an antivirulence agent.

## 1. Introduction

Polyphenols, among them flavonoids, exhibit a wide range of biological activities, including an antibacterial one [1,2,3].

However, their use in clinical practice is limited by low bioavailability, reducing therapeutic effects [4]. To increase bioavailability, various nanoformulations are used which enhance the solubility of polyphenols in the lipid and liquid phases. In the first case, liposomes loaded by polyphenols named nanophytosomes are used [5,6,7,8]. In the second case, compounds capable of forming more soluble complexes with polyphenols are applied. Such compounds include cyclodextrin [9,10], chitosan [11], and glycyrrhizin acid (GA) [12,13,14].

GA, isolated from roots of the plant *Glycyrriza Glabra*, is a natural glycoside belonging to the class of triterpenoids. Of all the complexants studied to date, GA exhibits the widest range of its own biological properties, such as antiviral, immunotropic, antiallergic, antitumor, and antiulcer activities [12,15,16], as well as neuroprotective [17], hepatoprotective [18], and hypoglycemic effects [19].

Various medicines have also been created on the basis of the GA and its derivatives. For example, glycyram (monoammonium glycyrrhizinate, GC) is an anti-inflammatory and antiallergic drug used in medical practice. Other glycyrrhizic acid derivatives are considered to be new antiviral and immune-modulating agents [20]. There are reports that glycyrrhizic acid is a drug candidate to treat COVID-19 [21,22].

However, in addition to a wide range of its own biological effects, GA has unique micelle-forming and complexing properties. These properties of GA are used to obtain an inclusion complex of GA with different medicines, allowing the creation of their new medicinal form. The unique ability of GA is associated with the peculiarities of its structure. GA has an amphiphilic nature and consists of a hydrophobic glycyrrhetic acid residue and two hydrophilic glucuronic acid molecules with a β-configuration of the glycosidic bond [4].

The GA has the ability to self-associate with the formation of a cyclic conformation, resulting in the creation of an intraspherical space, convenient for the production of an inclusion complex or a “host–guest” type complex where the host is GA and the guest is a medicinal compound (pharmakon) [12,13].

In this case, GA can serve as a drug delivery system and contributes to an increase in the absorption of the drug [12]. GA inclusion complexes were created for a range of compounds, such as cholesterol [23], lagochilin [24], paclitaxel [25,26], and others [13]. The formation of inclusion complexes makes it possible to increase the prolonging effect of medicinal compounds and their stability, as well as reduce the therapeutic load/dose. It was shown previously that the equimolar complex of GA with levomycetin significantly increased the survival of mice infected with *S. aureus*, *P. aeruginosa*, *P. vulgaris*, and *E. coli* compared to treatment with levomycetin or GA alone [27].

In connection with the above, we also used GA to obtain a complex with quercetin and studied its antimicrobial activity. Quercetin belongs to flavonoids, a secondary plant metabolite ubiquitously present in various fruits, vegetables, tea, and wine. At present, quercetin is the most studied flavonoid exhibiting a wide range of biological activities, such as antioxidant, anticarcinogen, antiviral, and neuroprotective ones [28,29]. Quercetin also shows good antimicrobial activity against a number of bacteria [30,31,32].

However, low bioavailability and instability of quercetin makes it difficult to use it in medical practice [29]. It was shown earlier that quercetin could form complexes with glycyram, a monoammonium salt of glycyrrhizic acid, in a ratio of 1:1 [33].

The production of such complexes with quercetin is aimed not only at increasing the bioavailability of the flavonoid, but also at slowing down its biodegradation, as it is known the quercetin is easily oxidized. It was previously found that quercetin–chitosan conjugates showed an enhancement of antioxidant and antimicrobial properties as well as of thermal degradability [11]. It was also demonstrated that quercetin–glycyrrhizin alginate nanogels successfully transport/deliver quercetin, preventing acute liver failure [34].

Moreover, we showed that the inclusion complex of GA with rutin (quercetin-3-O-rutinoside) exhibited lipid-lowering activity in a model of hyperlipidemia in vivo [14].

In this paper, we synthesized a complex of GA with quercetin in a ratio of 2:1, studied some of its physicochemical parameters, and showed its antimicrobial and antivirulent activities against *S. aureus* and the main toxin of these bacteria, α-hemolysin.

## 2. Materials and Methods

### 2.1. Materials

Glycyrrhizic acid (GA) was obtained from licorice root according to a previously known method and purified by the method in [35]. Quercetin was obtained on the basis of rutin from *Japanese sophora* flowers by acid hydrolysis (purity 98–99% by HPLC). 1,6-diphenyl-1,3,5-hexatriene (DPH), 1-(4-trimethylammoniumphenyl)-6-phenyl-1,3,5-hexatriene (TMA-DPH), α-hemolysin, acetone (chemically pure), ethanol (chemically pure), aqueous ammonia (25%), glacial acetic acid (chemically pure), sulfuric acid (chemically pure), potassium hydroxide (chemically pure), and acetonitrile were obtained from Merck (KGaA, Darmstadt, Germany). All microbiological media used in the study were supplied by Oxoid (Basingstoke, UK). All other reagents were purchased from POCH (Gliwice, Poland).

### 2.2. Preparation of GA–Quercetin Complex (GAQ)

GA (1646 g) was dissolved in 100 mL 50% C_2_H_5_OH. Then, 0.302 g of quercetin was added to the GA solution. The resulting mixture was intensively stirred with a magnetic stirrer for 8–9 h at room temperature. Then, the alcohol was evaporated using a rotary evaporator. The residue was freeze-dried. The yield of the final product was 87%. Tm. = 160–163 °C.

### 2.3. Measurement of UV–Visible Absorption Spectra

UV spectra were recorded from aqueous 50% C_2_H_5_OH solutions of compounds on a Shimadzu 1280 scanning spectrophotometer (Shimadzu, Kyoto, Japan) using a 1 cm quartz cuvette. The slit width was 5 nm, and reproducibility was ±1 nm. The measurements were carried out in the UV spectral range from 190 nm to 400 nm. Scanning speed was 400 nm/min. First, the spectrum of 50% ethanol as baseline was obtained, and then, the spectrum of the supramolecular complex GAQ (2:1) was obtained under the same conditions.

### 2.4. Measurement of IR Spectra

The IR spectra were taken in the form of tablets with KBr on a PerkinElmer IR-Fourier 2000 spectrometer (Perkin-Elmer, Buckinghamshire, UK) in the far IR region of the spectrum, i.e., 400–4000 cm^−1^. Scanning speed was 30 scans/s.

### 2.5. Bacterial Strain and Growth Conditions

*S. aureus* reference strain NCTC 5655 obtained from National Collection of Type Cultures (UK) was used in the study. Bacteria were grown overnight at 37 °C in Mueller Hinton (MH) broth with shaking at 200 rpm.

### 2.6. Antimicrobial Activity—Determination of Minimum Inhibitory Concentration (MIC) and Minimum Bactericidal Concentration (MBC)

The antibacterial activity of GAQ was assessed by monitoring the cell growth of *S. aureus* NCTC 5655 using the broth microdilution method, conducted according to the National Committee for Clinical Laboratory Standards. First, the compound was dissolved in ethanol, and the solution was added to Mueller Hinton broth (MHB) to give a final concentration of 7800 µg/mL. The samples were then serially two-fold diluted in bullion to obtain concentrations ranging from 3900 to 0.94 μg/mL in a 96-well microtiter plate with final volumes of 100 μL. Next, 100 μL of bacteria solution was injected into each well. The final bacteria cell concentration was 1 × 10^6^ colony forming units per mL (CFU/mL). The plates were incubated at 37 °C for 24 h for bacteria. The MIC value was determined to be the lowest concentration of an antibacterial agent that inhibited bacterial growth, as indicated by the absence of turbidity. The MBC value was determined to be the lowest concentration of antibacterial agents for which no bacterial growth on the plates was observed [36].

### 2.7. Hemolysis Study

The swine blood was centrifuged (837 g, 15 min, 4 °C); then, plasma and buffy coat were removed by aspiration. Erythrocytes were thrice washed with PBS, and then, 1% suspension was prepared in PBS. One milliliter of 1% suspension of erythrocytes was incubated for 30 min at 37 °C in the presence or absence of GAQ at the concentration range of 0.5–7.5 μg/mL. The 100 μL of supernatant from *S. aureus* (OD_600_ = 2.0) was added to each sample of erythrocytes. After incubation for 60 min at 37 °C, 0.5 mL of suspension was taken from every sample and mixed with 1 mL of PBS. To obtain 100% hemolysis, 1 mL of water was added to 0.5 mL of a control sample. All the samples were centrifuged, and absorbance of supernatants was measured using a Jasco V-770 spectrophotometer (Tokyo, Japan) at 540 nm [37].

### 2.8. Measurement of Erythrocytes Membrane Fluidity

The erythrocyte membrane ordering parameter was estimated by using a steady-state fluorescent polarization technique [38]. The suspension of erythrocytes (2 mL of 0.01% in PBS) was labeled with a fluorescent probe (DPH or TMA-DPH) at a concentration of 1 μM (10 min, 37 °C, in dark). The fluorescence measurements of samples in the absence or in the presence (0.005–0.075 µg/mL) of the studied compound were carried out at 37 °C using a PerkinElmer LS-55 (PerkinElmer, Buckinghamshire, UK) spectrofluorometer equipped with a fluorescence polarization device. The readings were taken at intervals of 2 s. The fluorescence anisotropy values (*r*) were automatically calculated using the fluorescence manager program according to Jablonski Equation (1) [38]:

(1) $$r=\frac{I_{VV}-GI_{VH}}{I_{VV}+2GI_{VH}}$$

where *I_VV_* and *I_VH_* are the fluorescence intensities emitted in vertical and horizontal directions while the exciting light beam is oriented vertically, respectively. The grating correction factor *G* = (*I_HV_*/*I_HH_*) corrects the polarizing effects of the monochromator. The excitation wavelengths were 348 nm (DPH) and 340 nm (TMA-DPH), and the fluorescence emission was measured at 426 nm for DPH and 430 nm for TMA-DPH. Based on the obtained values of *r*, the membrane ordering parameter was calculated using Equation (2):

(2) $$S=\frac{\sqrt{\left[1-2\left(\frac{r}{r_{0}}\right)+5{\left(\frac{r}{r_{0}}\right)}^{2}\right]}-1+\frac{r}{r_{0}}}{2\left(\frac{r}{r_{0}}\right)}$$

where *r*_0_ is the fluorescence anisotropy of DPH or TMA-DPH in the absence of any rotational motion of the probe [37]. The theoretical value of *r*_0_ of DPH and TMA-DPH is 0.4. Changes in the fluidity of the membrane of erythrocytes treated with the studied compounds in the concentration range of 0.005–0.075 µg/mL were determined based on the ratio of the ordering parameter of the sample in the presence of compound to the ordering parameter of the control (in the absence of the compound) [*S*/*S*_0_].

### 2.9. Fluorescence Studies of α-Hemolysin-GAQ Interaction

Fluorescence studies of α-hemolysin interaction with GAQ were made used the PerkinElmer LS-55B spectrofluorometer (PerkinElmer, Buckinghamshire, UK). Fluorescence signal (without and in the presence of GAQ) was monitored from α-hemolysin tryptophan’s (Trp) residues using excitation and emission wavelengths λ_exc._ = 295 nm and λ_em._ = 350 nm. Hemolysin was used at the final concentration of 100 nM and was titrated by the increasing concentration of GAQ in the concentration range of 0.154–0.770 µM (0.3–1.5 µg/mL, respectively). Since the GAQ can slightly absorb the excitation and emission wavelengths, raw fluorescence data (before taken to further analysis and calculations) were corrected on the inner filter effect using Equation (3) [39]:

(3) $$F_{cor}=F_{obs}\cdot 10^{\frac{\left(A_{exc.}+A_{em.}\right)}{2}}$$

where *A_exc._* and *A_em._* mean the GAQ absorbances at the excitation and emission wavelengths, whereas *F_cor._* and the *F_obs._* are the corrected and observed fluorescences, respectively.

Corrected fluorescence was used for calculations of physicochemical parameters characterizing α-hemolysin-GAQ interactions.

### 2.10. Statistical Analysis

The results are presented as mean ± SD. The level of significance was analyzed using a one-way ANOVA test. *p* < 0.05 and below was accepted as statistically significant. Statistical analysis was performed using Origin 8.5.1 software (Microcal Software Inc., Northampton, MA, USA).

## 3. Results and Discussion

Polyphenols in the form of extracts and infusions have been used as antimicrobial agents in traditional medicine and for food preservation since ancient times [40]. The mechanisms of polyphenols activities can be realized both at the level of whole bacteria and directly through interaction with isolated virulent factors. In the first case, there is a violation of the structure of the membranes, and as a result, there is an ionic imbalance, a disintegration membrane protein, an influence on the genetic apparatus, a repression of genes, and an inhibition of enzymatic activities and of energy metabolism [37,40]. In the second case, polyphenols bind to virulent factors, which leads to their inactivation and to the disruption of interaction with target cells.

Moreover, polyphenols can protect target cells against the action of toxins by inhibiting their interaction with membrane lipids, receptors [41], or their internalization [42], or by directly blocking their activity/permeability, which takes place in the case of pores-forming toxins [43,44,45].

However, a significant increase in bacterial resistance to antibiotics leads to the search for new medicines as well as to the modification of existing ones to improve their bioavailability and reduce drug resistance development [40,46].

### 3.1. Physicochemical Characteristic of a New Biomaterial (GAQ) Based on Quercetin and Glycyrrhizic Acid

**Figure 1:**
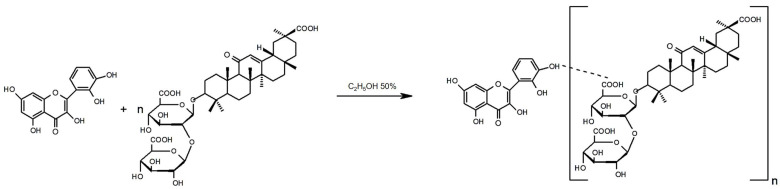
Scheme of synthesis of quercetin–glycyrrhizic acid GAQ complex (n = 2).

The synthesis of the GAQ (Figure 1) was carried out in an aqueous ethanol medium according to the method described in the Materials and Methods section. The complex was obtained by traditional mixing of solutions of origin substances with a molar ratio of components of 2:1. Next, some physicochemical characteristics of the complex and its optical parameters were studied.

**Figure 2:**
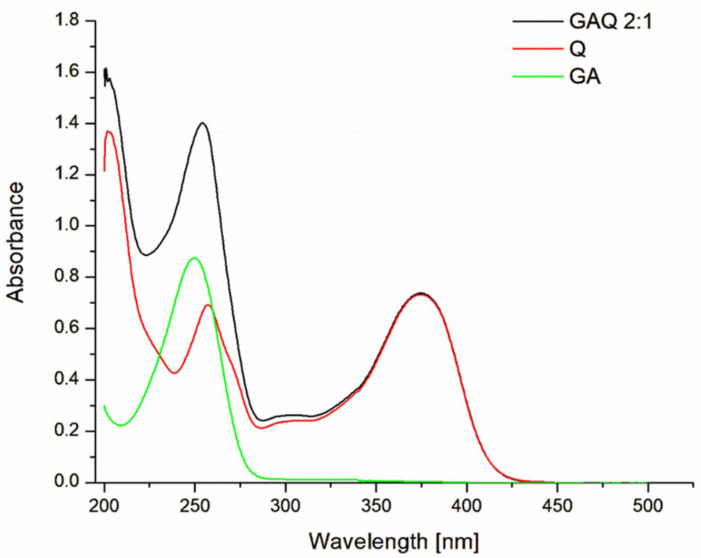
UV spectrum of GAQ (2:1), quercetin (Q), and glycyrrhizic acid (GA).

First, the UV spectra of the original chemical compounds and the obtained complex were studied (Figure 2). It was shown that quercetin has two main peak absorptions in the UV spectrum, with a maximum of 259 nm and 375 nm, respectively. The UV spectrum of the pure GA had a maximum of absorption band at 250 nm. In the UV spectrum of the supramolecular complex, the maximum corresponding to the GA was shifted by 5.6 nm into the long-wavelength region of the spectrum (“red shift”) and was observed at 256.6 nm, while the maximum absorption in the near region of the spectrum showed a “blue shift” at 2.5 nm. The absorption maximum of quercetin at 375 nm does not change. It should be noted that the binding of quercetin by GA, which has conjugated bonds in the aromatic ring of its molecule, was manifested by the “hyperchromic effect” of a complex maximum at 256.6 nm.

In the IR spectrum of quercetin (Table 1), the following characteristic vibration frequencies were found: in the frequency range of 3000–3600 cm^−1^, 1666–1613 cm^−1^, and 1000–1100 cm^−1^. In the IR spectrum of GA, the following characteristic vibration bands were observed: an intense band in the region of 3407 cm^−1^, 1715 cm^−1^, and 1654 cm^−1^, which refers to the stretching vibrations of the carbonyl conjugated with a double bond. In the IR spectrum of the GAQ (2:1) complex, a strong change in the shape of the spectra was observed, so in the region of 1600–1655 cm^−1^ and 1726 cm^−1^, there are three broad absorption bands, which can be explained by stretching vibrations of carboxyl carbonyl groups and the carbonyl adjacent to connections. The value of the stretching vibration of the carbonyl of the carboxyl groups of glucuronic acid in the GA molecule changes (1716 cm^−1^) in the resulting complex by 1726 cm^−1^, and the value of the carbonyl located next to the double bond changes (1656 cm^−1^) in the resulting complex by 1690 cm^−1^. This indicates that in this complex, an intermolecular hydrogen bond is formed between quercetin hydroxyls and carbonyls of carboxyl groups or GA carbonyls located next to the double bond in the aglycone part of the GA molecule. The broadening of the characteristic vibration frequencies in the region of 3400–3000 cm^−1^ indicates the formation of hydrogen bonds.

The formation of intermolecular hydrogen bonds during the formation of supramolecular complexes was also proven by IR spectroscopy of Lagochilin, Adenine, and Kinetin with GA and its salts [47,48,49].

The study of the physicochemical parameters of supramolecular complex of quercetin with GA showed that the melting point is 160–163 °C and the complex has a high solubility in water and 50% ethanol, but does not dissolve in organic solvents (Table 1).

### 3.2. Antibacterial and Antihemolytic Activity of GAQ

As an object for studying the antibacterial activity of the new biomaterial based on a combination of GA and quercetin (GAQ), we used the *S. aureus* strain NCTC 5655.

*S. aureus*, Gram-positive bacteria, is one of the most widespread and strong pathogens belonging to the genus Staphylococcus, causing both hospital- and community-acquired infections as skin inflammation, pneumonia, and even sepsis. *S. aureus* produces a plethora of virulence factors, including toxins. One of the main ones is α-hemolysin, which plays an important role in diseases caused by bacterial infection [50,51,52]. α-hemolysin refers to pore-forming toxins capable of exhibiting the typical β-hemolytic activity, causing a violation of the integrality of cells, and also breaking adherens junctions and compromising the cytoskeleton of epithelia and endothelia via activation metalloprotease [52].

To evaluate the microbiological effect of GAQ, the minimum inhibitory concentration (MIC) and minimum bactericidal concentration (MBC) were determined. The results obtained are presented in Table 2.

The minimum inhibitory concentration (MIC) of GAQ was at 487 µg/mL (250 µM), while at a concentration four times higher, it exhibited bactericidal activity (the MBC value is 1948 µg/mL; 1000 µM). It should be noted that the MIC value for the GAQ complex is within the range of the MIC values (250–1000 μg/mL) shown for quercetin for different strains of *S. aureus* [53], which indicates a good antibacterial activity of the studied complex.

It is known that antibacterial effects of flavonoids are determined not only by their capability of combating bacteria but also by their antivirulent activity, which includes reducing the production of virulent factors or neutralizing their activity.

Hemolysin is the main characteristic of the pathogenicity of *S. aureus* strains and has been considered to be a target for antivirulence/antitoxin strategy [54,55].

It has now been shown that flavonoids can actively neutralize the hemolytic activity of α-hemolysin *S. aureus*, causing a disturbance in the toxin structure [56,57,58]. Our earlier study revealed that quercetin at the concentration range of 10–80 µM inhibited sheep erythrocyte hemolysis induced by the *S. aureus*-produced α-hemolysin toxin; however, in that case, we showed another mechanism of the antihemolytic activity of quercetin, namely, the ability to increase the resilience of the erythrocyte membrane to the toxin [53].

Quercetin has also been demonstrated to inhibit α-hemolysin-induced hemolysis by preventing its secretion by bacteria [59].

In connection with the above, the antihemolytic activity of GAQ was studied using *S. aureus* strain NCTC 5655, which secretes only α-hemolysin. The obtained results are demonstrated below.

It should be emphasized that GAQ itself does not induce erythrocyte hemolysis. At all concentrations tested in the range of 0.5–7.5 μg/mL, erythrocyte hemolysis did not exceed the control hemolysis level of 1.95 ± 0.42%.

**Figure 3:**
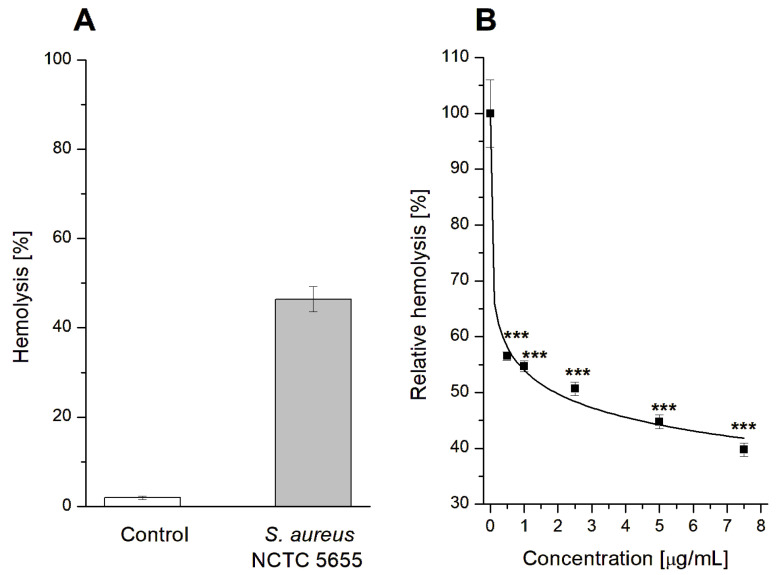
Inducing of hemolysis by α-hemolysin of *S. aureus* NCTC 5655 strain (**A**) and antihemolytic activity of GAQ biomaterial demonstrated as relative hemolysis (**B**). The data presented are the means ± SD. Lines are fits of logistic function to the data. Statistical significance was estimated using a one-way ANOVA test (results compared to control, *** *p* < 0.001).

Exposure of erythrocytes to the α-hemolysin of the *S. aureus* strain NCTC 5655 resulted in hemolysis of 46.41 ± 2.81% (Figure 3A), which was taken as 100% for the relative hemolysis analysis (Figure 3B). As shown in Figure 3B, GAQ inhibits bacterial-induced hemolysis in a concentration-dependent manner. At the highest concentration tested, 7.5 μg/mL, GAQ reduced hemolysis to 39.77 ± 1.19% compared to the control, which was taken as 100%. The logistic equation fitting to the data revealed the IC_50_ value for GAQ was 1.923 ± 0.255 µg/mL.

As was demonstrated above, GAQ possesses the ability to strongly decrease erythrocytes hemolysis induced by staphylococcal α-hemolysin. The observed antihemolytic effect of GAQ can be realized through two mechanisms: (i) interaction with target cells (erythrocytes); and/or (ii) interaction with α-hemolysin.

### 3.3. Influence of GAQ on the Structure of Erythrocytes Membrane

The first possible mechanism of the antihemolytic activity of GAQ may be connected with the interaction with the target cells—erythrocytes that increase their resistance to toxin (α-hemolysin). In this case, the interaction will be at the level of the cell membrane, which may change its structure under the influence of the compound. The action of GAQ on erythrocyte fluidity was analyzed using the fluorescent probes TMA-DPH and DPH, being located at different depths of the lipid bilayer. The TMA-DPH probe allows for the registering of changes in fluidity in the outer, hydrophilic part of the membrane, and of changes in DPH in the inner, hydrophobic region. The fluorescence anisotropy values were used to calculate the lipid order parameter (S) according to the Equation (2). The results are presented as the ratio (S/S_0_), where S_0_ and S represent lipid order parameter in the absence and in the presence of GAQ.

**Figure 4:**
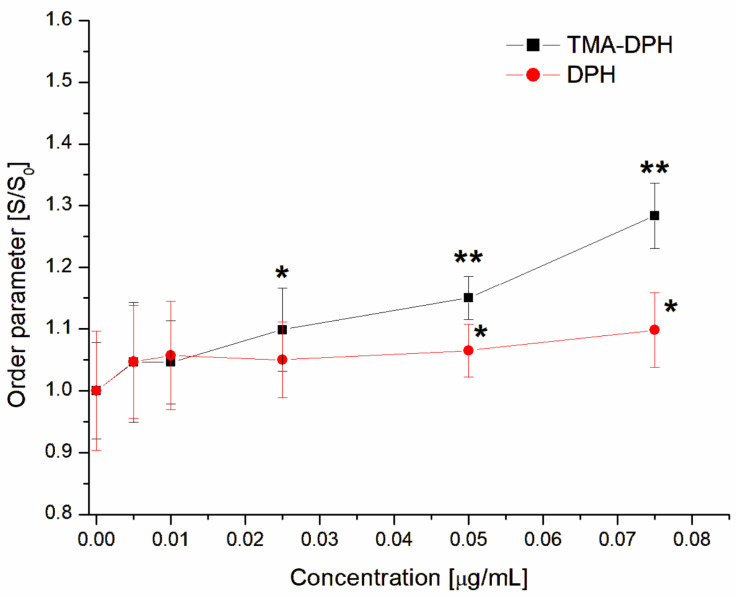
Changes in the order parameter S/S_0_ of erythrocytes membrane in the presence of GAQ. The data presented are the means ± SD. Statistical significance was estimated using a one-way ANOVA test (results compared to control, * *p* < 0.05; ** *p* < 0.01).

Figure 4 shows that the GAQ complex was able to increase the ordering parameter (S/S_0_) of erythrocyte membranes as determined by measuring changes in the fluorescence anisotropy of both probes. GAQ at a concentration of 0.075 µg/mL increases the ordering parameter (S/S_0_) from 1 (control) to 1.28 and 1.09 for TMA-DPH and DPH, respectively, indicating a large increase in stiffness in the hydrophilic part of the erythrocyte membrane compared to the hydrophobic part.

We previously showed that tannins, phenolic acid, and also the iodinated forms exhibited antihemolytic activity against α-hemolysin, reducing the fluidity of the hydrophilic part of sheep erythrocyte membrane [37,60]. It is known that the basis of the cytolytic action of the toxin is the formation of a pore from seven water-soluble monomers. This process is multistep and involves the binding of the monomer to the target cell membrane, the formation of nonlytic prepore as the result of oligomerization of monomers, and a subsequent transition to the channel [61,62]. It was shown that the liquid disorder phase was preferable for binding hemolysin to the membrane [63]. Therefore, we can assume that one of the mechanisms of the antihemolytic activity of the complex GAQ is to limit the binding/oligomerization of the toxin to the membrane of erythrocytes via a decrease in its fluidity. Previously, it was shown that quercetin exhibits antihemolytic activity regarding to a pore-forming cytotoxin pneumolysin (*S. pneumoniae*), and this effect is associated with the inhibition of oligomerization of the toxin in target cells [64].

### 3.4. Fluorescence Characterization of α-Hemolysin–GAQ Interaction

Another possible mechanism of the observed antihemolytic activity of GAQ can be the result of direct GAQ interaction with α-hemolysin, leading to the inhibition of its activity. In order to verify this hypothesis, the fluorescence analysis of α-hemolysin–GAQ interactions was conducted.

**Figure 5:**
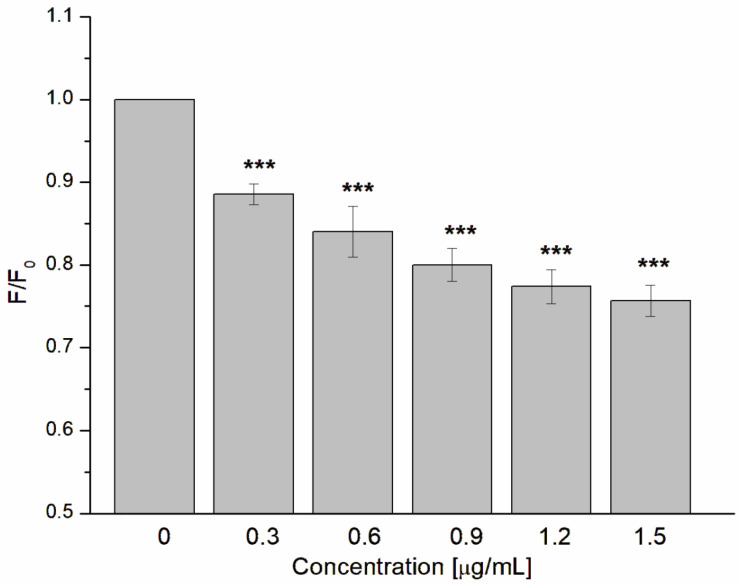
Relative fluorescence (F/F_0_) quenching of α-hemolysin Trp residues in the presence of GAQ. The data presented are the means ± SD. Statistical significance was estimated using a one-way ANOVA test (results compared to control, *** *p* < 0.001).

Fluorescence studies of protein–ligand interactions are the one of the most often used techniques due to their relative simplicity as well as the possibility to calculate some physicochemical parameters like Stern–Volmer or quenching constants that give basic information about interactions between guest and host molecules [65]. Fluorescence studies clearly demonstrated that GAQ possess a strong ability to interact with α-hemolysin, leading to a decrease in fluorescence signal, as presented below (Figure 5).

**Figure 6:**
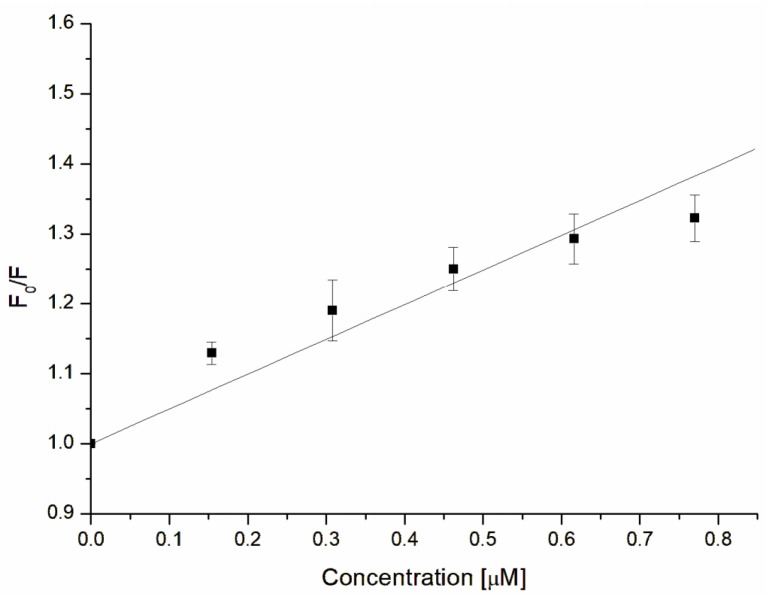
Stern–Volmer constant of fluorescence quenching in α-hemolysin Trp residues.

Observed fluorescence quenching can be the results of the complexation between the toxin and the studied compound (static quenching mechanism) and descends only from collisional encounters between used host–guest molecules (dynamic mechanism) [66]. In order to define which type of quenching occurred, the Stern–Volmer constant (K_SV_) and quenching constant (k_q_) were calculated based on the below Equation and Stern–Volmer plot (Figure 6):

(4) $$\frac{F_{0}}{F}=K_{SV}\left[Q\right]+1$$

where F_0_ and F are the fluorescence of α-hemolysin without and in the presence of GAQ, and K_SV_ is the Stern–Volmer constant.

The calculated Stern–Volmer constant describing the affinity of the quencher to the fluorophore was K_SV_ = (4.778 ± 0.585) × 10^5^ M^−1^. We postulate that the observed high affinity of GAQ to α-hemolysin can mainly descend from quercetin. For example, it was demonstrated that for the interaction of four studied flavonoids, i.e., quercetin, rutin, epicatechin, and catechin with bovine serum albumin, the quercetin possessed the highest Stern–Volmer constant [67]. Also, Soares described that flavonoids have a strong affinity to protein (α-amylase) [68]. It must be emphasized that quercetin, due to the fact that it is complexed with only two glycyrrhizic acid molecules, is not completely covered by glycyrrhizic acid molecules; thus, their ability to interact with proteins is still kept. On the other hand, glycyrrhizic acid can also participate in the interaction with α-hemolysin. It was demonstrated that the metabolite of glycyrrhizic acid, i.e., 18β-glycyrrhetinic acid, formed complexes with bovine serum albumin [69]; thus, the role of glycyrrhizic acid in GAQ interaction with α-hemolysin cannot also be excluded.

Using the Stern–Volmer constant and below Equation, the quenching constant (k_q_) was calculated:

(5) $$k_{q}=\frac{K_{SV}}{\tau_{0}}$$

where k_q_, K_SV_, and τ_0_ are the quenching constant, Stern–Volmer constant, and the fluorescence lifetime of fluorophore molecules (5 × 10^−9^ s), respectively.

The obtained k_q_ value ((9.56 ± 1.17) × 10^13^ M^−1^s^−1^) was larger than 2 × 10^10^ M^−1^s^−1^; therefore, it can be concluded that the fluorescence quenching of α-hemolysin tryptophan occurred because of the static mechanism with the complex formation between α-hemolysin and GAQ molecules. We suppose that complex formation between α-hemolysin and GAQ is also mainly controlled by the quercetin. As was described by Kameníková et al. [70] and Vaneková et al. [71], quercetin binds close to the tryptophan residues of serum albumin and quencher tryptophan fluorescence in static mechanisms.

**Figure 7:**
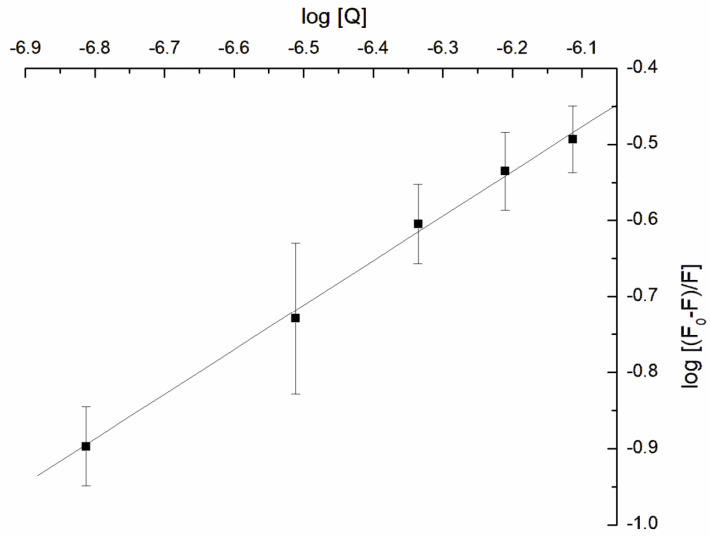
Double logarithmic plot of α-hemolysin fluorescence quenching by GAQ.

Based on the fluorescence measurement, the binding constant (logK_b_) was also calculated based on the double-logarithmic plot (Figure 7) drew according to Equation (6):

(6) $$\log\left[\frac{\left(F_{0}-F\right)}{F}\right]=logK_{b}+n\;log\;[Q]$$

where F_0_ and F are the fluorescences without and in the presence of GAQ, respectively, and the K_b_ is the binding constant.

According to Equation (4) and the above double logarithmic plot, the calculated logarithm of binding constants had the value logK_b_ = 3.135 ± 0.381. Molecules like warfarin, diazepam, or racemic ibuprofen bind to proteins like serum albumin with different binding constants [72], and the binding can be reversible for K_b_ within the range of 1–15 × 10^4^ M^−1^ (4–5 for logK_b_) [72,73]. In our experiments, the logK_b_ was below the mentioned range 4–5; thus, the α-hemolysin-GAQ interaction is stronger in comparison with the above-mentioned drugs’ interaction with albumin.

## 4. Conclusions

In this work, a new, complex biomaterial based on quercetin and glycyrrhizic acid was studied in order to evaluate its antibacterial and antihemolytic activity. Quercetin is a well-known flavonoid with a wide spectrum of biological activity that is limited by low polarity. In order to increase their bioavailability, the complex of quercetin with a natural glycoside, glycyrrhizic acid, belonging to the class of triterpenoids, was synthesized. GA exhibits a broad range of its own biological properties, e.g., antiviral or immunotropic. Based on obtained results, it can be concluded that the quercetin–glycyrrhizic acid complex (GAQ) possesses antibacterial activity against the *S. aureus* NCTC 5655 strain as well as demonstrates double antivirulence activity connected with the increase in erythrocyte membrane rigidity that inhibits the pore-formation in red blood cell membranes as well as strongly interacts with α-hemolysin-formed complexes with toxins that “switch-off” toxin oligomerization and formation of hemolytic pore.

Therefore, it can be concluded that the quercetin–glycyrrhizic acid complex can be a new potential antibacterial compound with activity that acts both on the bacteria as well as on the level of bacteria’s virulence factors.

## Figures and Tables

**Table 1:** Some physical/chemical parameters of the molecular complex of glycyrrhizic acid (GA) and quercetin (Q).

Complexes	Relation-ships	Melting Point. °C with Decomposition	Water	Ethanol 50%	Acetone	Hexane	Chloroform
GAQ	2:1	160–163	+	+	−	−	−
Compounds		IR spectrum, cm^−1^					
Q		3600−3000 (OH), 1666−1613 (C = O), 1100−1000 (C-O-C)					
GA		3407 (OH), 1715 (C = O), 1654 (=C-C = O)					
GAQ 2:1		1600, 1655, 1726 (C = O), 3400−3000 (OH)					

**Table 2:** Antibacterial activity of GAQ demonstrated as MIC and MBC values.

	MIC	MBC
GAQ [µg/mL]	487	1948
GAQ [µM]	250	1000

## Data Availability

Not applicable.

## References

[1] Daglia M. (2012). Polyphenols as antimicrobial agents. Curr. Opin. Biotechmol..

[2] Cushnie T.P.T., Lamb A.J. (2011). Recent Advances in Understanding the Antibacterial Properties of Flavonoids. Int. J. Antimicrob. Agents.

[3] Xie Y., Chen J., Xiao A., Liu L. (2017). Antibacterial Activity of Polyphenols: Structure-Activity Relationship and Influence of Hyperglycemic Condition. Molecules.

[4] Tolstikov G.A., Baltina L.A., Shul’ts E.E., Pokrovsky A.G. (1997). Glycyrrhizic acid. Russ. J. Bioorganic Chem..

[5] Babazadeh A., Ghanbarzadeh B., Hamishehkar H. (2017). Phosphatidylcholine-rutin complex as a potential nanocarrier for food. J. Funct. Food..

[6] Salcedo C.L., Frias M.A., Cutro A.C., Nazareno M.A., Disalvo E.A. (2014). Antiradical activity of gallic acid included in lipid interphases. Biochim. Biophys. Acta.

[7] Naziris S., Sekowski S., Olchowik-Grabarek E., Buczkowski A., Balcerzak Ł., Chrysostomou V., Pispas S., Małecka M., Bryszewska M., Ionov M. (2023). Biophysical interactions of mixed lipid-polymer nanoparticles incorporating curcumin: Potential as antibacterial agent. Biomater. Adv..

[8] Amer S.S., Mamdouh W., Nasr M., ElShaer A., Polycarpou E., Abdel-Aziz R.T.A., Sammour O.A. (2022). Quercetin loaded cosm-nutraceutical electrospun composite nanofibers for acne alleviation: Preparation, characterization and experimental clinical appraisal. Int. J. Pharm..

[9] Pinho E., Soares G., Henriques M. (2015). Evaluation of antibacterial activity of caffeic acid encapsulated by β-cyclodextrins. J. Microencapsul..

[10] Ilyich T.V., Kovalenia T.A., Lapshina E.A., Stępniak A., Pałecz B., Zavodnik I.B. (2021). Thermodynamic parameters and mitochondrial effects of supramolecular complexes of quercetin with β-cyclodextrins. J. Mol. Liq..

[11] Torres E., Marin V., Aburto J., Beltran H.I., Shirai K., Villanueva S., Sandoval G. (2012). Enzymatic modification of chitosan with quercetin and its application as antioxidant edible films. Prikl. Biokhim. Mikrobiol..

[12] Su X., Wu L., Hu M., Dong W., Xu M., Zhang P. (2017). Glycyrrhizic acid: A promising carrier material for anticancer therapy. Biomed. Pharmacother..

[13] Salyutina O.Y., Polyakov N.E. (2019). Glycyrrhizic acid as a multifunctional drug carrier—From physicochemical properties to biomedical applications: A modern insight on the ancient drug. Int. J. Pharm..

[14] Baratov Q., Mustafakulov M., Matchanov A., Vypova N., Yakubova R., Tagayalieva N. (2021). Glycyrrhizic acid and its derivatives as the carries for the poorly soluble flavonoids. Int. J. Disaster Recovery Bus. Contin..

[15] Batiha G.E.S., Beshbishy A.M., El-MLeeh A., Abdel-Daim M.M., Devkota H.P. (2020). Traditional Uses, Bioactive Chemical Constituents, and Pharmacological and Toxicological Activities of *Glycyrrhiza glabra* L. (Fabaceae). Biomolecules.

[16] Wen Y., Chen H., Zhang L., Wu M., Zhang F., Yang D., Shen J., Chen J. (2021). Glycyrrhetinic acid induces oxidative/nitrative stress and drives ferroptosis through activating NADPH oxidases and iNOS, and depriving glutathione in triple-negative breast cancer cells. Free Radic. Biol. Med..

[17] Cherng J.M., Lin H.J., Hung M.S., Lin Y.R., Chan M.H., Lin J.C. (2006). Inhibition of nuclear factor kappaB is associated with neuroprotective effects of glycyrrhizic acid on glutamate-induced excitotoxicity in primary neurons. Eur. J. Pharmacol..

[18] Huo X., Meng X., Zhang J., Zhao Y. (2020). Hepatoprotective effect of different combinations of 18α-and 18β-Glycyrrhizic acid against CCl_4_-induced liver injury in rats. Biomed. Pharmacother..

[19] Zhang W., Li T., Zhang X.-J., Zhu Z.-Y. (2020). Hypoglycemic effect of glycyrrhizic acid, a natural non-carbohydrate sweetener, on streptozotocin-induced diabetic mice. Food Funct..

[20] Baltina L., Kondratenko R. (2021). Glycyrrhizic Acid Derivatives as New Antiviral and Immune Modulating Agents. Curr. Bioact. Compd..

[21] Sun Z., He G., Huang N., Thilakavathy K., Lim J.C.W., Kumar S.S., Xiong C. (2021). Glycyrrhizic acid: A natural plant ingredient as a drug candidate to treat COVID-19. Front. Pharmacol..

[22] Zhao Z., Xiao Y., Xu L., Liu Y., Jiang G., Wang W., Li B., Zhu T., Tan Q., Tang H. (2021). Glycyrrhizic Acid Nanoparticles as Antiviral and Anti-inflammatory Agents for COVID-19 Treatment. ACS Appl. Mater. Interfaces.

[23] Gluschenko O.Y., Polyakov N.E., Leshina T.V. (2011). NMR Relaxation Study of Cholesterol Binding with Plant Metabolites. Appl. Magn. Reson..

[24] Matchanov A.D., Dalimov D.N., Zainutdinov U.N., Vypova N.L., Islamov A.K., Bekpolatova B.M. (2017). Preparation and Physicochemical and Biological Properties of Molecular Associates of Lagochilin and Lagochirsine with Glycyrrhizic Acid and its Monoammonium Salt. Chem. Nat. Compd..

[25] Yang F.H., Zhang Q., Liang Q.Y., Wang S.Q., Zhao B.X., Wang Y.T., Cai Y., Li G.F. (2015). Bioavailability Enhancement of Paclitaxel via a Novel Oral Drug Delivery System: Paclitaxel-Loaded Glycyrrhizic Acid Micelles. Molecules.

[26] Hussain M. (2019). Molecular dynamics simulations of glycyrrhizic acid aggregates as drug-carriers for paclitaxel. Curr. Drug Deliv..

[27] Kondratenko R.M., Baltina L.A., Mustafina S.R., Ismagilova A.F., Zarudii F.S., Davydova V.A., Bazekin G.V., Suleimanova G.F., Tolstikov G.A. (2003). Complex compounds of glycyrrihizic acid with antimicrobial drugs. Pharm. Chem. J..

[28] Boots A.W., Haenen G.R.M.M., Bast A. (2008). Health effects of quercetin: From antioxidant to nutraceutical. Eur. J. Pharmacol..

[29] Azeem M., Hanif M., Mahmood K., Ameer N., Rahman F., Chugtai S., Abid U. (2023). An insight into anticancer, antioxidant, antimicrobial, antidiabetic and anti-inflammatory effects of quercetin: A review. Polym. Bull..

[30] Adamczak A., Ożarowski M., Karpiński T.M. (2020). Antibacterial Activity of Some Flavonoids and Organic Acids Widely Distributed in Plants. J. Clin. Med..

[31] Xie Y., Yang W., Tang F., Chen X., Ren L. (2015). Antibacterial Activities of Flavonoids: Structure-Activity Relationship and Mechanism. Curr. Med. Chem..

[32] Luo P., Liu D., Li J. (2020). Pharmacological perspective: Glycyrrhizin may be an efficacious therapeutic agent for COVID-19. Int. J. Antimicrob. Agents.

[33] Yakovishin L.A., Korzh E.N. (2019). Molecular complex of quercetin with glycyram. AIP Conf. Proc..

[34] Zhao F.-Q., Wang G.-F., Xu D., Zhang H.-Y., Cui Y.-L., Wang Q.-S. (2021). Glycyrrhizin mediated liver-targeted alginate nanogels delivers quercetin to relieve acute liver failure. Int. J. Biol. Macromol..

[35] Kondratenko R.M., Baltina L.A., Mikhailova L.R., Danilov V.T., Gabbasov T.M., Murinov Y.I., Tolstikov G.A. (2005). Preparation of glycyrrhizic acid and its practically important salts from licorice root extract. Chem. Pharm. J..

[36] Sekowski S., Naziris N., Chountoulesi M., Olchowik-Grabarek E., Czerkas K., Veiko A., Abdulladjanova N., Demetzos C., Zamaraeva M. (2023). Interaction of *Rhus typhina* tannin with lipid nanoparticles: Implication for the formulation of a tannin-liposome hybrid biomaterial with antibacterial activity. J. Funct. Biometer..

[37] Olchowik-Grabarek E., Sekowski S., Bitiucki M., Dobrzynska I., Shlyonsky V., Ionov M., Burzynski P., Roszkowska A., Swiecicka I., Abdulladjanova N. (2020). Inhibition of interaction between *Staphylococcus aureus* α-hemolysin and erythrocytes membrane by hydrolysable tannins: Structure-related activity study. Sci. Rep..

[38] Olchowik E., Lotkowski K., Mavlyanov S., Abdullajanova N., Ionov M., Bryszewska M., Zamaraeva M. (2012). Stabilization of erythrocytes against oxidative and hypotonic stress by tannins isolated from sumac leaves (*Rhus typhina* L.) and grape seeds (*Vitis vinifera* L.). Cell Mol. Biol. Lett..

[39] Molina-Bolivar J.A., Carnero Ruiz C., Galisteo-Gonzales F., Medona-O’ Donnell M., Parra A. (2016). Simultaneous presence of dynamic and sphere action component in the fluorescence quenching of human serum albumin by diphthaloylmaslinic acid. J. Lumin..

[40] Kumar H., Bhardwaj K., Cruz-Martins N., Nepovimova E., Oleksak P., Dhanjal D.S., Bhardwaj S., Singh R., Chopra C., Verma R. (2021). Applications of Fruit Polyphenols and Their Functionalized Nanoparticles Against Foodborne Bacteria: A Mini Review. Molecules.

[41] Thakur P., Chawla R., Narula A., Goel R., Arora R., Sharma R.K. (2016). Anti-hemolytic, hemagglutination inhibition and bacterial membrane disruptive properties of selected herbal extracts attenuate virulence of Carbapenem Resistant *Escherichia coli*. Microb. Pathog..

[42] Morinaga N., Yahiro K., Noda M. (2010). Resveratrol, a natural polyphenolic compound, inhibits cholera toxin-induced cyclic AMP accumulation in Vero cells. Toxicon.

[43] Tombola F., Campello S., De Luca L., Ruggiero P., Del Giudice G., Papini E., Zoratti M. (2003). Plant polyphenols inhibit VacA, a toxin secreted by the gastric pathogen *Helicobacter pylori*. FEBS Lett..

[44] Ostroumova O.S., Efimova S.S., Schagina L.S. (2011). 5- and 4′-Hydroxylated flavonoids affect voltage gating of single alpha-hemolysin pore. Bioch. Biophys. Acta.

[45] Olchowik-Grabarek E., Sekowski S., Mies F., Bitiucki M., Swiecicka I., Abdulladjanova N., Shlyonsky V., Zamaraeva M. (2023). Electrophysiological and spectroscopic investigation of hydrolysable tannins interaction with α-hemolysin of *S. aureus*. Bioelectrochemistry.

[46] Gupta P.D., Birdi T.J. (2017). Development of botanicals to combat antibiotic resistance. J. Ayurveda Integr. Med..

[47] Matchanov A.D., Zaynutdinov U.N., Islamov A.K., Vipova N.L., Tashpulatov F.N., Matchanov U.D. (2017). Supramolecular Complexes of Glycyrrhizic Acid, its Monoammonium Salt with Diterpenoid Lagochilin and their Hemostatic Activity. Biochem. Ind. J..

[48] Matchanov A.D., Zaynutdinov U.N., Islamov A.K., Vypova N.L., Tashpulatov F.N., Esanov R.S., Matchanov U.D., Sobirova F.A., Khakberdiev S.M. (2018). Synthesis and hemostatic activity of supramolecular complexes lagochilin. Int. J. Dev. Res..

[49] Matmuratov B.Y.A., Madraximova S.D., Esanov R.S., Eshchanov E.U., Matchanov A.D., Xakberdiev S.M. (2021). Supramolecular complexes of kinetin and adenin with glycyrrhizin acid and its monoammonium salt. Ann. Phytomed. Int. J..

[50] Los F.C.O., Randis T.M., Raffi R.V., Ratner A.J. (2013). Role of pore-forming toxins in bacterial infectious diseases. Microbiol. Mol. Biol. Rev..

[51] Oliveira D., Borges A., Simões M. (2018). *Staphylococcus aureus* Toxins and Their Molecular Activity in Infectious Diseases. Toxins.

[52] Cheung G.Y.C., Bae J.S., Otto M. (2021). Pathogenicity and virulence of *Staphylococcus aureus*. Virulence.

[53] Veiko A.G., Olchowik-Grabarek E., Sekowski S., Roszkowska A., Lapshina E.A., Dobrzynska I., Zamaraeva M., Zavodnik I.B. (2023). Antimicrobial activity of quercetin, naringenin and catechin: Flavonoids inhibit *Staphylococcus aureus*-induced hemolysis and modify membranes of bacteria and erythrocytes. Molecules.

[54] Escajadillo T., Nizet V. (2018). Pharmacological Targeting of Pore-Forming Toxins as Adjunctive Therapy for Invasive Bacterial Infection. Toxins.

[55] Divyakolu S., Chikkala R., Ratnakar K.S., Sritharan V. (2019). Hemolysins of *Staphylococcus aureus* —An Update on Their Biology, Role in Pathogenesis and as Targets for Anti-Virulence Therapy. Adv. Infect. Dis..

[56] Qiu J., Niu X., Dong J., Wang D., Wang J., Li H., Luo M., Li S., Feng H., Deng X. (2012). Baicalin protects mice from *Staphylococcus aureus* pneumonia via inhibition of the cytolytic activity of α-hemolysin. J. Infect. Dis..

[57] Wang J., Zhou X., Liu S., Li G., Shi L., Dong J., Li W., Deng X., Niu X. (2015). Morin hydrate attenuates *Staphylococcus aureus* virulence by inhibiting the self-assembly of α-hemolysin. J. Appl. Microbiol..

[58] Silva L.N.A., Da Hora G.C.A., Soares T.A., Bojer M.S., Ingmer H., Macedo A.J., Trentin D.S. (2017). Myricetin protects Galleria mellonella against *Staphylococcus aureus* infection and inhibits multiple virulence factors. Sci. Rep..

[59] He S., Deng Q., Liang B., Yu F., Yu X., Guo D., Liu X., Dong H. (2021). Suppressing Alpha-Hemolysin as Potential Target to Screen of Flavonoids to Combat Bacterial Coinfection. Molecules.

[60] Olchowik-Grabarek E., Mies F., Sekowski S., Dubis A., Laurent P., Zamaraeva M., Swiecicka I., Shlyonsky V. (2022). Enzymatic Synthesis and Characterization of Aryl Iodides of Some Phenolic Acids with Enhanced Antibacterial Properties. Biochim. Biophys. Acta Biomembr..

[61] Berube B.J., Wardenburg J.B. (2013). *Staphylococcus aureus* α-Toxin: Nearly a Century of Intrigue. Toxins.

[62] Sugawara T., Yamashita D., Kato K., Peng Z., Ueda J., Kaneko J., Kamio Y., Tanaka Y., Yao M. (2015). Structural basis for pore-forming mechanism of staphylococcal α-hemolysin. Toxicon.

[63] Schwiering M., Brack A., Stork R., Hellmann N. (2013). Lipid and phase specificity of α-toxin from S. aureus. Biochim. Biophys. Acta.

[64] Lv Q., Zhang P., Quan P., Cui M., Liu T., Yin Y., Chi G. (2020). Quercetin, a pneumolysin inhibitor, protects mice against *Streptococcus pneumoniae* infection. Microb. Pathog..

[65] Bose A. (2016). Interaction of tea polyphenols with serum albumins: A fluorescence spectroscopic analysis. J. Lumin..

[66] Lakowicz J.R. (2006). Principles of Fluorescence Spectroscopy.

[67] Papadopoulou A., Green R.J., Frazier R.A. (2005). Interaction of Flavonoids with Bovine Serum Albumin: A Fluorescence Quenching Study. J. Agric. Food Chem..

[68] Soares S., Mateus N., de Freitas V. (2007). Interaction of different polyphenols with bovine serum albumin (BSA) and human salivary alpha-amylase (HSA) by fluorescence quenching. J. Agric. Food Chem..

[69] Zhou N., Liang Y.-Z., Wang P. (2007). 18_-Glycyrrhetinic acid interaction with bovine serum albumin. J. Photochem. Photobiol. A Chem..

[70] Kameníková M., Furtmüller P.G., Klacsová M., Lopez-Guzman A., Toca-Herrera J.L., Vitkovská A., Devínsky F., Mučaji P., Nagy M. (2017). Influence of quercetin on the interaction of gliclazide with human serum albumin—Spectroscopic and docking approaches. Luminescence.

[71] Vaneková Z., Hubčík L., Toca-Herrera J.L., Furtmüller P.G., Valentová J., Mučaji P., Nagy M. (2019). Study of Interactions between AmLodipine and Quercetin on Human Serum Albumin: Spectroscopic and Modeling Approaches. Molecules.

[72] Dufour C., Dangles O. (2005). Flavonoid-serum albumin complexation: Determination of binding constants and binding sites by fluorescence spectroscopy. Biochim. Biophys. Acta.

[73] Suryawanshi V.D., Walekar L.S., Gore A.H., Anbhule P.V., Kolekar G.B. (2016). Spectroscopic analysis on the binding interaction of biologically active pyrimidine derivative with bovine serum albumin. J. Pharm. Anal..

